# Author response to: Comment on: Sexual harassment, sexual assault and rape by colleagues in the surgical workforce, and how women and men are living different realities: observational study using NHS population-derived weights

**DOI:** 10.1093/bjs/znad441

**Published:** 2024-01-19

**Authors:** Christopher T Begeny, Homa Arshad, Tamzin Cuming, Daljit K Dhariwal, Rebecca A Fisher, Marieta D Franklin, Philippa C Jackson, Greta M McLachlan, Rosalind H Searle, Carrie Newlands

**Affiliations:** Faculty of Health and Life Sciences, Department of Psychology, University of Exeter, Exeter, UK; Barts Bone and Joint Health, Barts NHS Trust, Royal London Hospital, London, UK; Department of Surgery, Homerton University Hospital, London, UK; Oxford University Hospitals NHS Foundation Trust, Nuffield Department of Surgical Sciences, University of Oxford, John Radcliffe Hospital, Oxford, UK; School of Medical Sciences, Division of Medical Education, University of Manchester, Manchester, UK; Department of Trauma and Orthopaedics, Liverpool University Hospitals NHS Foundation Trust, Liverpool, UK; Department of Surgery, North Bristol NHS Trust, Bristol, UK; Department of Surgery, Frimley Health Foundation Trust, Frimley, UK; Adam Smith Business School, University of Glasgow, Glasgow, UK; School of Biosciences and Medicine, University of Surrey, Guildford, UK

*Dear Editor*

The authors appreciate Krishnan *et al*. for their comments on Begeny *et al*.^1^ and fully agree with the points raised.

Going forward, it will indeed be critical to examine issues of sexual harassment and sexual violence in a more comprehensive manner, including from an intersectional perspective that will need to encompass several social identities, reflecting race and (racialized) ethnicity, class, age, sexual orientation, neurodiversity, parental/career status, relationship/marital status, nationality, and immigration status, for example, as well as gender, going beyond the typical focus on (primarily cis, endo) women and men. Similarly, going forward, it will be important to carefully integrate and build upon insights derived from past work on intersectionality theory, gendered races, racialized genders, intersectional invisibility, and other relevant frameworks.

Taking these next steps is very important and will require investment from key organizations (the General Medical Council (GMC), the National Health Service (NHS), etc.). They will be an essential source of support, such as for the recruitment of large samples of workforce members into studies that are, by design, able to robustly examine issues of sexual harassment and sexual violence from an intersectional perspective.

The authors recognize that the study by Begeny *et al*.^1^ did not have the external support needed to generate a more comprehensive set of insights. Thus, Begeny *et al*.^1^ stands not as a ‘final point’ to these issues, but it represents a notable step forward; a step that has helped bring key organizations to the table, to recognize the gravity of these issues and, hopefully, spur the motivation and investment that is ultimately needed to take the critical next steps.

The authors agree that issues of intersectionality could have been addressed in the article and regret not giving these issues the attention they deserve. The authors’ accompanying report^2^, which was released in parallel and looks at the wider implications for healthcare, does briefly explore these important issues. Still, more should have been done in the Begeny *et al*.^1^ article itself to draw attention to issues of intersectionality.

Finally, while space is limited, the authors want to highlight two other key points that Krishnan *et al*. raise. First, they note the importance of examining how sexual harassment and violence affect staff well-being and patient outcomes. The authors could not agree more. Second, they note that ‘Women from diverse backgrounds could face other abuses, including bullying, microaggression, and disparagement, in addition to sexual violence’ and that ‘This would make them more vulnerable and therefore less likely to report abuse’. This highlights an important facet of workplace inequality and a valuable point of focus for future research. While there is research on myriad forms of workplace inequality (for example, in evaluations, pay, hiring, promotions, access to mentorship, backlash effects, glass-cliff effects, motherhood penalties, etc.)—including some that examines how they manifest at intersections of key social identities, including among multiply marginalized women—how the presence of and potential for these inequalities interact with one’s experiences with (and tendency to report) sexual harassment and sexual violence is still a largely open question. This more broadly highlights how imperative it is for organizational leaders to understand the full scope of inequality that persists in their organizations, including its intersectional nature, and to respond in ways that actively help to address this issue.
